# Are gastrointestinal symptoms associated with higher risk of Mortality in COVID-19 patients? A systematic review and meta-analysis

**DOI:** 10.1186/s12876-022-02132-0

**Published:** 2022-03-07

**Authors:** Yang Wang, Yimin Li, Yifan Zhang, Yun Liu, Yulan Liu

**Affiliations:** 1 Department of Gastroenterology, Peking University People’s Hospital, No. 11, Xizhimen South Street, Xicheng District, Beijing, 100044 People’s Republic of China; 2 Clinical Center of Immune-Mediated Digestive Diseases, Peking University People’s Hospital, No. 11, Xizhimen South Street, Xicheng District, Beijing, 100044 People’s Republic of China; 3 Department of Rheumatology & Immunology, Beijing Key Laboratory for Rheumatism Mechanism and Immune Diagnosis (BZ0135), Peking University People’s Hospital, No. 11, Xizhimen South Street, Xicheng District, Beijing, 100044 People’s Republic of China

**Keywords:** Gastrointestinal symptom, COVID-19, Mortality, Prognosis

## Abstract

**Background:**

Gastrointestinal symptoms have been reported in patients with COVID-19. Several clinical investigations suggested that gastrointestinal symptoms were associated with disease severity of COVID-19. However, the relevance of gastrointestinal symptoms and mortality of COVID-19 remains largely unknown. We aim to investigate the relationship between gastrointestinal symptoms and COVID-19 mortality.

**Methods:**

We searched the PubMed, Embase, Web of science and Cochrane for studies published between Dec 1, 2019 and May 1, 2021, that had data on gastrointestinal symptoms in COVID-19 patients. Additional literatures were obtained by screening the citations of included studies and recent reviews. Only studies that reported the mortality of COVID-19 patients with/without gastrointestinal symptoms were included. Raw data were pooled to calculate OR (Odds Ratio). The mortality was compared between patients with and without gastrointestinal symptoms, as well as between patients with and without individual symptoms (diarrhea, nausea/vomiting, abdominal pain).

**Results:**

Fifty-three literatures with 55,245 COVID-19 patients (4955 non-survivors and 50,290 survivors) were included. The presence of GI symptoms was not associated with the mortality of COVID-19 patients (OR=0.88; 95% CI 0.71–1.09; *P*=0.23). As for individual symptoms, diarrhea (OR=1.01; 95% CI 0.72–1.41; *P*=0.96), nausea/vomiting (OR=1.16; 95% CI 0.78–1.71; *P*=0.46) and abdominal pain (OR=1.55; 95% CI 0.68–3.54; *P*=0.3) also showed non-relevance with the death of COVID-19 patients.

**Conclusions:**

Gastrointestinal symptoms are not associated with higher mortality of COVID-19 patients. The prognostic value of gastrointestinal symptoms in COVID-19 requires further investigation.

**Supplementary Information:**

The online version contains supplementary material available at 10.1186/s12876-022-02132-0.

## Background

The occurrence and rapid spread of novel coronavirus (SARS-CoV-2)-infected pneumonia (COVID-19) since December, 2019, has brought troublesome challenges to worldwide public health [1]. Globally, as of February 25, 2022, there have been 430,257,564 confirmed cases of COVID-19, including 5,922,049 deaths, reported to the WHO. In response to the alarming levels of its spread, severity and death threat of COVID-19, the WHO issued a statement of Public Health Emergency of International Concern on January 30, 2020 and further declared COVID-19 a pandemic on March 11, 2020 [2].

The most frequent symptoms in COVID-19 patients are respiratory manifestations. However, emerging studies have found that gastrointestinal (GI) symptoms including diarrhea, nausea/vomiting and abdominal pain, are also commonly observed in patients with COVID-19, with a prevalence of up to 31.9% [3, 4].

As the major receptor of SARS-CoV-2, angiotensin-converting enzyme 2, is also expressed in the gastrointestinal tract [5]. Early evidence has identified gastrointestinal infection of SARS-CoV-2 via immunofluorescent [6]. Intriguingly, several case-control studies and meta-analysis suggested that COVID-19 patients with GI symptoms might be at a higher risk of clinical deterioration [7, 8]. Physicians are also anxious to find out whether GI symptoms in patients with COVID-19 indicate a higher probability of death. In the first few months of COVID-19 pandemic, Mao et al. performed a meta-analysis and found that COVID-19 patients with GI symptoms tended to have higher prevalence of death (OR (odds ratio) = 1.21) but without statistical significance (*P* = 0.52) [8]. The question remains controversial due to the limited number of studies and population at that time. Now with the numerous emerging publications reporting the characteristics and outcomes of COVID-19 patients, there is a pressing need to determine the role of GI symptoms in the prognosis of COVID-19. Hence, this meta-analysis is conducted to investigate the relationship between GI symptoms and the mortality of COVID-19 patients.

## Methods

### Search strategy and selection criteria

We searched PubMed, Embase, Web of Science and Cochrane databases on May 1, 2021 for articles published from Dec 1, 2019, using the keywords combination of “COVID-19”, “SARS-CoV-2”, “2019 novel coronavirus”, “2019-nCoV”, “coronavirus disease 2019”, “coronavirus disease-19”, “severe acute respiratory syndrome coronavirus” and “novel Coronavirus 2019” for COVID-19, and “gastrointestinal”, “vomiting”, “vomit”, “nausea”, “diarrhoea”, “diarrhea”, “appetite”, “anorexia”, “abdominal”, “abdomen”, “digestive” and “alimentary” for GI symptoms. The reference lists of relevant reviews, meta-analysis and included literatures were also screened manually to identify additional articles that might be missed in the database search. Search records were managed with EndNote (version X7) for excluding duplicates and further literature screening. Preferred Reporting Items for Systematic Reviews and Meta-Analyses (PRISMA) guidelines were followed. The protocol of this meta-analysis has been registered with the International Prospective Register of Systematic Reviews (PROSPERO, registration number CRD42020197032).

The eligibility for inclusion of literatures were determined by three authors (YW, YmL and YZ) independently, and dissonance were discussed with another author (YL) and subsequently resolved via consensus. Articles reporting the mortality of COVID-19 patients with and without GI symptoms respectively were considered eligible for inclusion. Preprint studies without peer-review were excluded due to potential misinformation. The following literatures were excluded at title and abstract screening: reviews, meta-analysis, guidelines, case reports, letter, comment, editorial, protocol, clinical research with less than 20 patients, basic research and non-relevant literatures. Then full-text review was performed to exclude articles without needed data and those written in languages other than English.

### Data extraction and definitions

Three authors (YW, YmL and YZ) independently extracted the data, and dissonance were resolved with another author (YL) by discussion and consensus. The following variables were extracted: first author, study location, number of patients, basic characteristics of study population, mortality of COVID-19 patients with and without GI symptoms, respectively (Additional file 1).

For studies only reporting individual symptoms such as diarrhea, nausea, vomiting and abdominal pain, “GI symptom” was defined as the most common one of these digestive symptoms. For studies reporting either nausea or vomiting but not nausea/vomiting, “nausea/vomiting” was defined as the more frequent one of the two symptoms.

### Assessment of study quality

For included studies, Newcastle-Ottawa Scale (NOS) was used for the assessment of quality. NOS is a quality assessment tool for observational studies that has been endorsed by the Cochrane Collaboration [9, 10]. The studies were considered as high quality if they scored > 6 points, moderate quality if they scored 5 or 6 points, and poor quality if they scored < 5 points.

### Data synthesis and statistical analysis

To ensure the accuracy of the results, analysis were performed by two authors (YW and YmL) independently. Dissonance was resolved by discussion. To evaluate the risk of mortality associated with GI symptoms, OR with 95% confidence intervals (CI) were calculated by the Cochrane Review Manager program (RevMan 5.3, Denmark) following the Mantel-Haenszel method. The heterogeneity of included literatures was detected by *I*^2^ statistic.

Subgroup analysis was performed according to the study location, severity of disease, patient age and population size. Funnel-plot and Egger’s test were used to investigate the possibility of publication bias. *P* < 0.05 for Egger’s test was considered significant bias. If publication bias was indicated, trim-and-fill method was used for adjusting OR. A sensitivity analysis was also performed by omitting each study using the *meta* package in R, version 4.0.2.

## Results

### Search results and study characteristics

**Fig. 1:**
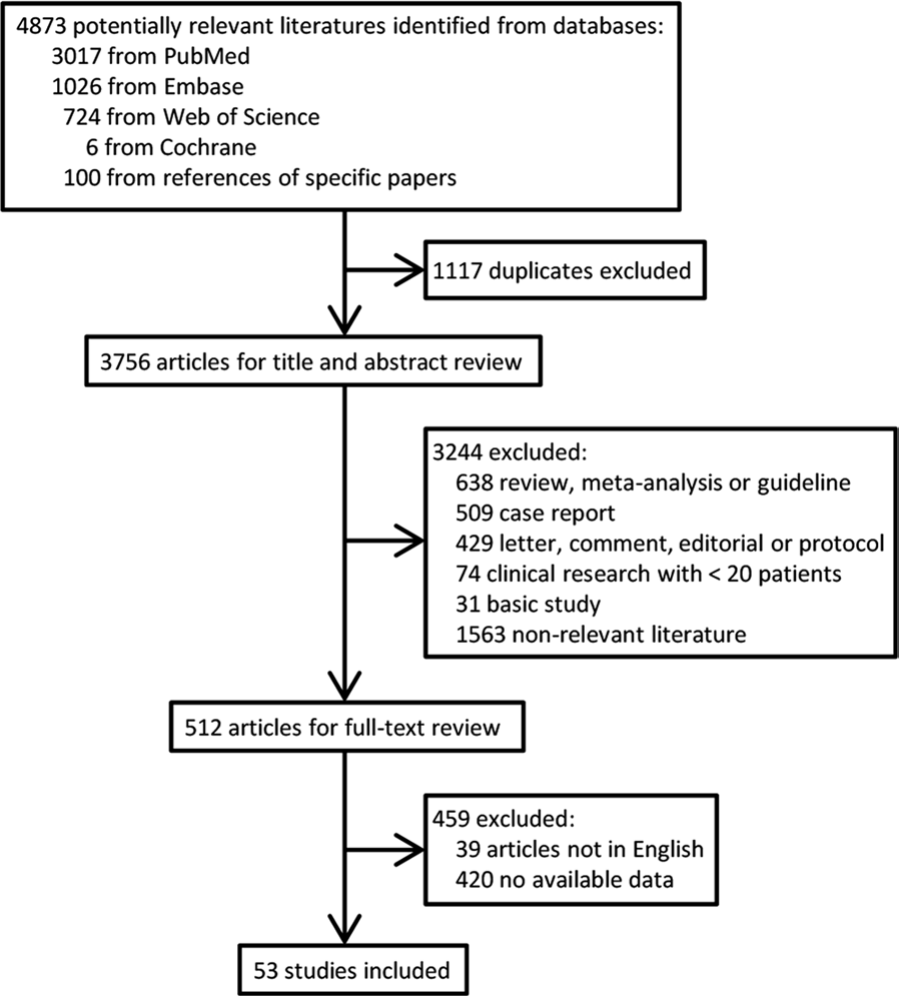
Flow chart showing the flow of study selection

The study selection process is depicted in Fig. 1. A total of 4,873 records were initially identified. After removal of duplicates, 3,756 remained. After screening by titles/abstracts and full-text review, 53 studies [11–63] were finally included for data analysis.

The characteristics of the included studies are shown in Table 1. Of the 53 included studies with a total of 55,245 patients, 21 were carried out in China, 12 in USA and 20 in other countries. One and four studies included pediatric and geriatric patients, respectively. Seven studies investigated COVID-19 combined with other disease history including chronic liver disease, cancer, kidney transplantation and interstitial lung disease. Six studies included critically ill patients. All papers were considered high quality with NOS score > 6.

**Table 1:** Characteristics of the studies included for meta-analysis

No. of study	First author	Study location	No. of patients	Age (year)	Male	Special patient population	NOS score
1	Alizadehsani R et al.	Iran	123	45	62	None	8
2	An P et al.	China	205	54	122	None	8
3	Atalla E et al.	USA	111	87	23	Older patients	7
4	Caillard S et al.	France	243	62	162	Kidney transplant recipients	7
5	Chadalavada P et al.	USA	84	60	52	None	8
6	Chen R et al.	China	1077	59	532	None	9
7	Chen T et al.	China	274	62	171	Critically ill patients	7
8	Comoglu Ş et al.	Turkey	1086	48	563	None	8
9	Crespo M et al.	Spain	414	62	265	Kidney transplant recipients	7
10	Doganci S et al.	Turkey	397	57	200	None	8
11	Du H et al.	China	182	6	120	Pediatric patients	7
12	Elimian K et al.	Nigeria	3215	36	2293	None	8
13	Ferm S et al.	USA	877	59	534	None	8
14	Gayam V et al.	USA	408	67	231	African-Americans	7
15	Ghoshal U et al.	India	252	40	204	None	8
16	Hajifathalian K et al.	USA	1059	61	611	None	8
17	Huang H et al.	China	49	37	23	Patients with pre-existing ILD	7
18	Jiang Y et al.	China	281	70	143	Older severe patients	7
19	Jin X et al.	China	651	46	331	None	9
20	Kang M et al.	Korea	118	59	52	None	8
21	Kim D et al.	USA	867	57	473	Patients with chronic liver disease	7
22	Lanthier N et al.	Belgium	50	88	NA	Geriatric patients	7
23	Laszkowska M et al.	USA	2804	66	1565	None	8
24	Leal T et al.	Portugal	201	71	113	Symptomatic patients	7
25	Liang J et al.	China	109	65	57	Patients with cancer	7
26	Liu J et al.	China	29,393	47	15,501	None	8
27	Livanos A et al.	USA	634	61	369	None	8
28	Luo S et al.	China	1411	54	895	None	8
29	Ma X et al.	China	467	44	289	None	8
30	Montazeri M et al.	Iran	611	56	377	None	8
31	Moura D et al.	Brazil	400	56	225	None	8
32	Nobel Y et al.	USA	278	NA	145	None	7
33	Pan L et al.	China	204	53	107	None	9
34	Peng X et al.	China	49	63	17	Critically ill patients	7
35	Ramachandran P et al.	USA	150	57	83	None	8
36	Redd W et al.	USA	318	63	174	None	9
37	Renelus B et al.	USA	734	68	379	None	8
38	Russell B et al.	UK	156	65	90	Patients with cancer	7
39	Schettino M et al.	Italy	190	65	127	None	8
40	Shang H et al.	China	564	59	286	None	8
41	Soares R et al.	Brazil	1152	NA	494	None	8
42	Sulaiman T et al.	Iraq	140	45	100	None	8
43	Tsibouris P et al.	Greece	61	70	34	None	8
44	Vena A et al.	Italy	275	71	183	None	8
45	Villanego F et al.	Spain	1011	60	635	Kidney transplant recipients	7
46	Vrillon A et al.	France	52	90	34	Older adults	7
47	Wan, Y et al.	China	230	48	129	None	8
48	Wang Z et al.	China	59	67	38	Critically ill patients	7
49	Yang X et al.	China	52	60	35	Critically ill adults	7
50	Zhang J et al.	China	663	56	321	None	7
51	Zhang L et al.	China	409	65	234	Severe COVID-19 patients	7
52	Zhou F et al.	China	191	56	72	None	7
53	Zhou Z et al.	China	254	50	115	None	9

NOS, Newcastle-Ottawa Scale; ILD, interstitial lung disease; COVID-19, corona virus disease 2019

Clinical features and outcomes of COVID-19 patients are listed in Table 2. Of the 55,245 patients, 4,955 non-survivors were reported. A total of 8,535 patients had GI symptoms. Individual GI symptoms included diarrhea (1,341 reported in 10,983 patients), nausea/vomiting (525 reported in 7,175 patients) and abdominal pain (92 reported in 5,012 patients). The cumulative incidences of GI symptom, diarrhea, nausea/vomiting and abdominal pain in COVID-19 patients were 25%, 16%, 7.5% and 3.6%, respectively.

**Table 2:** Clinical outcomes and manifestations of the patients included for meta-analysis

No. of study	Author	No. of patients	No. of death	No. of GI symptom	No. of diarrhea	No. of nausea/vomiting	No. of abdominal pain
1	Alizadehsani R et al.	123	15 (12.2%)	11 (8.9%)	NA	NA	NA
2	An P et al.	205	6 (2.9%)	79 (38.5%)	NA	NA	NA
3	Atalla E et al.	111	48 (43.2%)	8 (7.2%)	8 (7.2%)	2(1.8%)	NA
4	Caillard S et al.	243	43 (17.7%)	96 (39.5%)	96 (39.5%)	NA	NA
5	Chadalavada P et al.	84	11 (13.1%)	44 (52.4%)	NA	NA	NA
6	Chen R et al.	1077	85 (7.9%)	359 (33.3%)	NA	NA	NA
7	Chen T et al.	274	113 (41.2%)	77 (28.1%)	77 (28.1%)	24(8.8%)	19(6.9%)
8	Comoglu Ş et al.	1086	38 (3.5%)	78 (7.2%)	78 (7.2%)	NA	NA
9	Crespo M et al.	414	109 (26.3%)	152 (36.7%)	NA	NA	NA
10	Doganci S et al.	397	34 (8.6%)	292 (73.6%)	NA	NA	NA
11	Du H et al.	182	1 (0.5%)	20 (11.0%)	9 (4.9%)	7(3.8%)	7(3.8%)
12	Elimian K et al.	3215	295 (9.2%)	132 (4.1%)	132 (4.1%)	103(3.2%)	20(0.6%)
13	Ferm S et al.	877	208 (23.7%)	219 (25.0%)	NA	NA	NA
14	Gayam V et al.	408	132 (32.4%)	111 (27.2%)	NA	NA	NA
15	Ghoshal U et al.	252	5 (2.0%)	26 (10.3%)	NA	NA	NA
16	Hajifathalian K et al.	1059	147 (13.9%)	349 (33.0%)	NA	NA	NA
17	Huang H et al.	49	9 (18.4%)	3 (6.1%)	3 (6.1%)	1 (2.0%)	NA
18	Jiang Y et al.	281	114 (40.6%)	33 (11.7%)	33 (11.7%)	13 (4.6%)	NA
19	Jin X et al.	651	1 (0.2%)	74(11.4%)	NA	NA	NA
20	Kang M et al.	118	6 (5.1%)	54 (45.8%)	54(45.8%)	NA	NA
21	Kim D et al.	867	121 (14.0%)	181 (20.9%)	181 (20.9%)	175 (20.2%)	NA
22	Lanthier N et al.	50	26 (52.0%)	15 (30.0%)	12 (24.0%)	3 (6.0%)	3 (6.0%)
23	Laszkowska M et al.	2804	542 (19.3%)	1084 (38.7%)	NA	NA	NA
24	Leal T et al.	201	55 (27.4%)	60 (29.9%)	NA	NA	NA
25	Liang J et al.	109	23 (21.1%)	26 (23.9%)	26 (23.9%)	10 (9.2%)	5 (4.6%)
26	Liu J et al.	29,393	711 (2.4%)	2289 (7.8%)	NA	NA	NA
27	Livanos A et al.	634	151 (23.8%)	299 (47.2%)	NA	NA	NA
28	Luo S et al.	1411	66 (4.7%)	183 (13.0%)	NA	NA	NA
29	Ma X et al.	467	16 (3.4%)	25 (5.4%)	25(5.4%)	NA	NA
30	Montazeri M et al.	611	104 (17.0%)	155 (25.4%)	NA	NA	NA
31	Moura D et al.	400	89 (22.3%)	133 (33.3%)	NA	NA	NA
32	Nobel Y et al.	278	9 (3.2%)	97 (34.9%)	56 (20.1%)	63 (22.7%)	NA
33	Pan L et al.	204	36 (17.6%)	103 (50.5%)	NA	NA	NA
34	Peng X et al.	49	16 (32.7%)	22 (44.9%)	11 (22.4%)	15 (30.6%)	3(6.1%)
35	Ramachandran P et al.	150	58 (38.7%)	31 (20.7%)	NA	NA	NA
36	Redd W et al.	318	32 (10.1%)	195 (61.3%)	NA	NA	NA
37	Renelus B et al.	734	237 (32.3%)	231 (31.5%)	NA	NA	NA
38	Russell B et al.	156	34 (21.8%)	25 (16.0%)	NA	NA	NA
39	Schettino M et al.	190	41 (21.6%)	138 (72.6%)	NA	NA	NA
40	Shang H et al.	564	51 (9.0%)	157 (27.8%)	157 (27.8%)	NA	NA
41	Soares R et al.	1152	456 (39.6%)	126 (10.9%)	126 (10.9%)	NA	NA
42	Sulaiman T et al.	140	12 (8.6%)	78 (55.7%)	NA	NA	NA
43	Tsibouris P et al.	61	16 (26.2%)	11 (18.0%)	11 (18.0%)	4 (6.6%)	2 (3.3%)
44	Vena A et al.	275	120 (43.6%)	14 (5.1%)	14(5.1%)	11(4.0%)	NA
45	Villanego F et al.	1011	220 (21.8%)	323 (31.9%)	NA	NA	NA
46	Vrillon A et al.	52	17 (32.7%)	17 (32.7%)	NA	NA	NA
47	Wan, Y et al.	230	6 (2.6%)	49 (21.3%)	49 (21.3%)	NA	NA
48	Wang Z et al.	59	41 (69.5%)	22 (37.3%)	22 (37.3%)	4 (6.8%)	NA
49	Yang X et al.	52	32 (61.5%)	2 (3.8%)	NA	2 (3.8%)	NA
50	Zhang J et al.	663	25 (3.8%)	61 (9.2%)	61 (9.2%)	31 (4.7%)	5 (0.8%)
51	Zhang L et al.	409	102 (24.9%)	91 (22.2%)	91 (22.2%)	50 (12.2%)	28 (6.8%)
52	Zhou F et al.	191	54 (28.3%)	9 (4.7%)	9 (4.7%)	7 (3.7%)	NA
53	Zhou Z et al.	254	16 (6.3%)	66 (26.0%)	NA	NA	NA
Cumulative incidence				25%	16%	7.5%	3.6%

GI, gastrointestinal; NA, not available

### Association of GI symptoms with the mortality of COVID-19

**Fig. 2:**
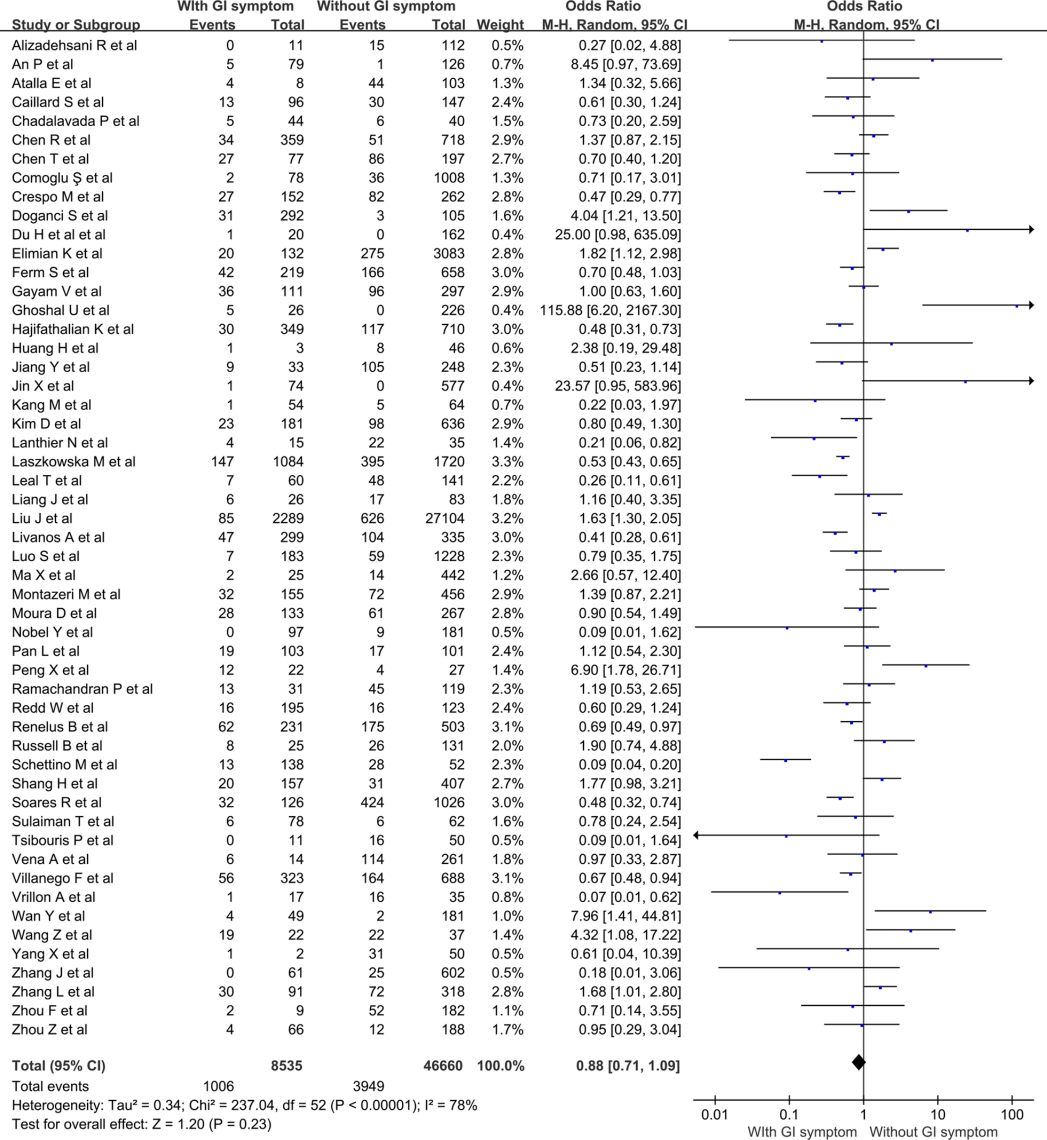
Forest plots showing pooled odds ratio of gastrointestinal symptoms associated with the mortality of COVID-19

As shown in Fig. 2, presence of GI symptom was found to have no significant association with the mortality of COVID-19 (OR = 0.88; 95% CI 0.71–1.09; *P* = 0.23). There was substantial heterogeneity among the 53 studies included (*I*^2^ = 78%, P < 0.001).

**Fig. 3:**
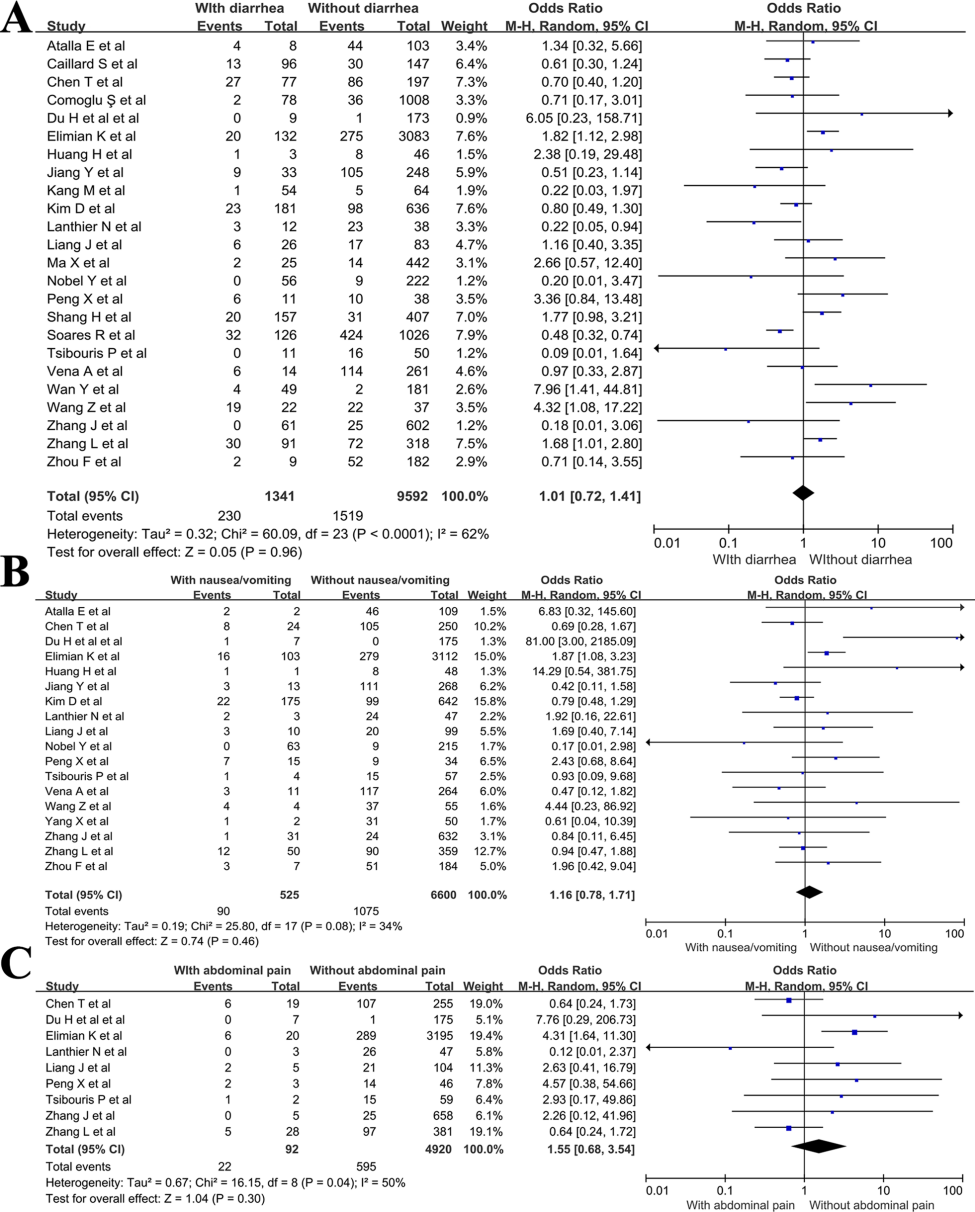
Forest plots showing pooled odds ratio of (**A**) diarrhea, (**B**) nausea/vomiting and (**C**) abdominal pain associated with the mortality of COVID-19

For individual GI symptoms, there were 24 studies reporting on diarrhea (Fig. 3a), 18 on nausea/vomiting (Fig. 3b), and 9 on abdominal pain (Fig. 3c). The pooled OR of diarrhea was 1.01 (95% CI 0.72–1.41; *P* = 0.96), of nausea/vomiting was 1.16 (95% CI 0.78–1.71; *P* = 0.46), and of abdominal pain was 1.55 (95% CI 0.68–3.54; *P* = 0.3). No substantial heterogeneity was found in the studies included for the analysis of nausea/vomiting and abdominal pain (*I*^2^ = 34% and 50%, respectively). While moderate heterogeneity was observed for diarrhea (*I*^2^ = 62%).

### Subgroup Analysis

Since substantial heterogeneity was observed for GI symptom, we performed subgroup analysis to explore the source of heterogeneity. As shown in Table 3, as for studies conducted in different locations, the heterogeneity was moderate in the subgroups of Asia and America (*I*^2^ = 54.7% and 42.7%, respectively). The heterogeneity remained significant in the subgroups of Europe and other continents (*I*^2^ = 77% and 87.8%, respectively). Notably, data from Asian studies indicated that GI symptom was a significant risk factor for the death of COVID-19 patients (OR = 1.43, *P* = 0.01). On the contrary, American and European studies showed that GI symptom was associated with a lower mortality risk (OR = 0.64 and 0.4, respectively; *P* < 0.01 for both). The study location seemed to be a major source of heterogeneity. Meanwhile heterogeneity remained substantial in the other subgroups in terms of disease severity and population size, and GI symptom had no significant relevance with mortality in these subgroups. Nevertheless, in subgroup analysis Asian literatures indicate GI symptom is a significant risk factor for the mortality of COVID-19 (OR > 1, *P* < 0.05). Meanwhile European and American studies suggest that GI symptom is a significant protective factor (OR < 1, P < 0.05). The possible explanation for these contradictory observations has always been controversial. In an European research Crespo et al. found that patients with gastrointestinal COVID-19 phenotype recovered more frequently [19]. Several studies from the USA described that COVID-19 patients with GI symptoms were younger, with less comorbidity [33, 37]. Therefore, we further reviewed the literatures in our analysis. Among the literatures that had data on the age of patients with/without GI symptoms, all of the European and American literatures (n = 8 of 8, 100%) [15, 19, 33, 34, 37, 45, 46, 49] reported that patients with GI symptoms were younger than those without. And OR values were < 1 in 7 [15, 19, 33, 34, 37, 46, 49] of these 8 literatures. On the other hand, 7 [12, 16, 18, 25, 29, 36, 57] of 13 Asian studies [12, 16, 18, 25, 29, 30, 36, 38, 40, 43, 50, 52, 57] reported that patients with GI symptoms were older, and OR values were > 1 in 6[12, 16, 25, 29, 36, 57] of these 7 studies. It turns out that the studies carried out in different locations vary in the characteristics of included patients, especially in the age of patients with/without GI symptoms. Given that old age is an important risk factor for the death of COVID-19 patients [14], the discordance in the findings in different study locations may be due to the differences in the age of included patients.

**Table 3:** Subgroup analysis based on study location, type of participants and population size

Subgroups	No. of studies	No. of patients	OR and P value for mortality of different symptoms				
			GI symptom	*I* ^2^ for GI symptom	Diarrhea	Nausea/vomiting	Abdominal pain
1. Study location							
1.1 Asia	28	39,501 (72%)	1.43, *P*=0.01	54.7%	1.32, *P*=0.21	1.3, *P*=0.36	1.07, *P*=0.85
Sub-subgroups for studies in Asia:							
1.1.1 GI group older than non-GI group	7	32,894	2.43, *P*<0.01	66.9%			
1.1.2 GI group younger than non-GI group	6	3048	1.2, *P*=0.29	15.8%			
1.2 America	12	8324 (15%)	0.64, *P*<0.01	42.7%	0.81, *P*=0.37	0.84, *P*=0.8	NA
Sub-subgroups for studies in America:							
1.2.1 GI group older than non-GI group	NA	NA	NA	NA			
1.2.2 GI group younger than non-GI group	5	3990	0.55, *P*<0.01	30.6%			
1.3 Europe	10	2653 (5%)	0.4, *P*<0.01	77%	0.51, *P*=0.07	0.7, *P*=0.51	0.61, *P*=0.76
Sub-subgroups for studies in Europe:							
1.3.1 GI group older than non-GI group	NA	NA	NA	NA			
1.3.2 GI group younger than non-GI group	3	805	0.23, *P*<0.01	84%			
1.4 Other	3	4767(8%)	0.92, *P*=0.83	87.8%	0.93, *P*=0.92	NA	NA
2. Only include critically ill patients?							
3.1 Yes	6	1124(2%)	1.42, *P*=0.36	74.6%	1.3, *P*=0.45	0.92, *P*=0.71	0.75, *P*=0.45
3.2 No	47	54,121 (98%)	0.83, *P*=0.1	78.3%	0.92, *P*=0.69	1.37, *P*=0.28	2.67, *P*=0.05
3. Population size							
4.1 <500	36	7436 (13%)	0.93, *P*=0.66	72.1%	1.05, *P*=0.82	1.19, *P*=0.51	1.04, *P*=0.92
4.2 >=500	17	47,809 (87%)	0.84, *P*=0.25	85.7%	0.94, *P*=0.83	1.16, *P*=0.68	NA
4. Average age of GI group and non-GI group							
4.1 GI group older than non-GI group	8	33,294 (60%)	1.89, *P*=0.02	69%			
4.2 GI group younger than non-GI group	14	7843 (14%)	0.61, *P*=0.01	80%			
4.3 Unknown	31	14,058 (26%)	0.89, *P*=0.36	64%			

OR, odds ratio; GI, gastrointestinal; NA, not available

To demonstrate the above finding, we explored the age related sub-analysis by study region. As shown in Table 3, the studies in Asia, America and Europe were divided into subgroups based on the age difference of included patients. Consistent with the age distribution, GI symptom was found to be a significant risk factor for mortality (OR > 1 and *P* < 0.05) in the subgroup that GI group was older than non-GI group (Table 3, subgroup 1.1.1). Meanwhile, GI symptom was a significant protective factor (OR < 1 and *P* < 0.05) in the subgroups that GI group was younger than non-GI group (Table 3, subgroup 1.2.2 and subgroup 1.3.2). Since the number of studies in each subgroup was quite small, we also performed subgroup analysis based on the age difference of included patients irrespective of the study region. As shown in Table 3, three additional subgroups were determined: [1] subgroup 4.1 included the studies in which the patients in GI group were older than those in non-GI group; [2] subgroup 4.2 included the studies in which the patients in GI group were younger than those in non-GI group; [3] subgroup 4.3 included the studies without available information on the age of patients in GI and non-GI group. Interestingly, in subgroup 4.1, GI symptom was a significant risk factor for mortality (OR = 1.89, *P* = 0.02). On the contrary, in subgroup 4.2, GI symptom was a significant protective factor for mortality (OR = 0.61, *P* = 0.01). This finding further supports our deduction that the difference in the age of GI and non-GI groups leads to the discordance in the findings in different study locations. The forest plots of these additional subgroup analysis are available in the Supplementary Material (Additional file 2: Figs. S1 to S6).

As for individual symptoms including diarrhea, nausea/vomiting and abdominal pain, none of these symptoms showed significant correlation with mortality (*P* of OR > 0.05 for all subgroups).

### Age stratification analysis

To further explore the relationship of GI symptom with mortality in different age groups, we performed additional age stratification analysis. As shown in Table 4, we stratified the studies into 5 groups according to the average age of the study population: 0–39, 40–49, 50–59, 60–69 and 70~. We expected that with the increased population age, GI symptom might be a risk factor from mortality. However, the actual results were contrary to our expectation: in younger populations (0–39, 40–49 and 50–59), GI symptom seemed to be a risk factor (OR > 1) while in older populations (60–69 and 70~) GI symptom showed a significant protective effect (OR < 1 and *P* < 0.05). The forest plots are available in Additional file 2. To clarify this finding, we reviewed the included studies again. We found that the average age of GI group was older than non-GI group in most studies (83.3%) with younger populations (40–49); meanwhile the average age of GI group was younger than non-GI group in all studies (100%) with older populations (60–69 and 70~). Overall, we supposed that the potential patients selection bias in the age of patients with/without GI symptom led to the discordance in the results.

**Table 4:** Age stratification analysis

Age stratification	No. of studies	OR and 95% CI of GI symptom for mortality	P value of OR	No. of studies that average age: GI group >non GI group	No. of studies that average age: GI group <non-GI group
0–39	3	2.36 [0.88; 6.33]	0.088	NA	NA
40–49	8	2.22 [0.96; 5.11]	0.061	5 (83.3%)	1 (16.7%)
50–59	15	1.09 [0.85; 1.40]	0.517	3 (33.3%)	6 (66.7%)
60–69	18	0.71 [0.54; 0.95]	0.02	0 (0%)	6 (100%)
70~	7	0.40 [0.21; 0.76]	0.006	0	1 (100%)

OR, odds ratio; CI, confidence interval; GI, gastrointestinal; NA, data are not available because the studies in the subgroups did not report the age information of patients with/without GI symptoms

### ***Publication bias analysis***

**Fig. 4:**
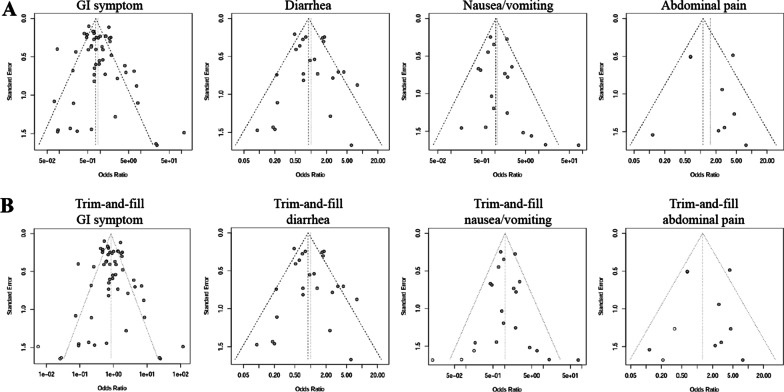
Funnel plots for evaluation of publication bias. **A** shows the funnel plots of gastrointestinal symptoms, diarrhea, nausea/vomiting and abdominal pain. **B** shows the funnel plots after trim-and-fill method

The funnel plots (Fig. 4 a) were found to be slightly asymmetric for GI symptom and nausea/vomiting. As shown in Table 5, Egger’s regression test also revealed publication bias for both factors (*P* = 0.05 and 0.04, respectively). Thus we performed trim-and-fill method to estimate missing studies (Fig. 4b) so as to make pooled OR more reliable. The P values of Egger’s test were > 0.05 after trim-and-fill adjustment (Table 5), indicating that the publication bias was reduced. After adjustment for presumed un-published reports after trim-and-fill analysis (Table 5), GI symptoms and individual symptoms remained uncorrelated with the death risk of COVID-19 (OR close to 1, and *P* > 0.05 for all).

**Table 5:** Publication bias analysis

	No. of included literatures	OR	*P* value for OR	P value of Egger’s test
Original data				
GI symptom	53	0.88	0.23	0.05
Diarrhea	24	1.01	0.96	0.55
Nausea/vomiting	18	1.16	0.46	0.04
Abdominal pain	9	1.55	0.3	0.61
After trim-and-fill				
GI symptom	56	0.84	0.11	0.82
Diarrhea	24	1.01	0.96	0.55
Nausea/vomiting	21	1.02	0.91	0.9
Abdominal pain	11	1.28	0.51	0.94

OR, odds ratio; GI, gastrointestinal

### Sensitivity analysis

As depicted in Table 6, the results of GI symptom showed good stability with all OR estimates (ranging from 0.86 to 0.92) within the 95% CI of pooled OR. The OR estimates of diarrhea, nausea/vomiting and abdominal pain were also stable when omitting one study at a time. All of the estimates showed no statistical significance, which were also in accordance with the major conclusion that the relationship of GI symptoms and mortality was not significant.

**Table 6:** Sensitivity analysis

	OR and *P* value for mortality of different symptoms			
Study omitted	GI symptom	Diarrhea	Nausea/vomiting	Abdominal pain
Omitting Alizadehsani R et al.	0.88, *P*=0.25	NA	NA	NA
Omitting An P et al.	0.86, *P*=0.17	NA	NA	NA
Omitting Atalla E et al.	0.87, *P*=0.22	1, *P*=1	1.13, *P*=0.55	NA
Omitting Caillard S et al.	0.89, *P*=0.28	1.04, *P*=0.81	NA	NA
Omitting Chadalavada P et al.	0.88, *P*=0.25	NA	NA	NA
Omitting Chen R et al.	0.87, *P*=0.2	NA	NA	NA
Omitting Chen T et al.	0.89, *P*=0.27	1.04, *P*=0.83	1.23, *P*=0.33	1.91, *P*=0.17
Omitting Comoglu Ş et al.	0.88, *P*=0.25	1.02, *P*=0.9	NA	NA
Omitting Crespo M et al.	0.9, *P*=0.31	NA	NA	NA
Omitting Doganci S et al.	0.86, *P*=0.15	NA	NA	NA
Omitting Du H et al. et al	0.87, *P*=0.18	0.99, *P*=0.96	1.08, *P*=0.63	1.42, *P*=0.42
Omitting Elimian K et al.	0.86, *P*=0.16	0.96, *P*=0.82	1.05, *P*=0.82	1.05, *P*=0.9
Omitting Ferm S et al.	0.88, *P*=0.28	NA	NA	NA
Omitting Gayam V et al.	0.88, *P*=0.23	NA	NA	NA
Omitting Ghoshal U et al.	0.86, *P*=0.15	NA	NA	NA
Omitting Hajifathalian K et al.	0.9, P=0.32	NA	NA	NA
Omitting Huang H et al.	0.87, *P*=0.21	1, *P*=0.98	1.12, *P*=0.57	NA
Omitting Jiang Y et al.	0.89, *P*=0.29	1.05, *P*=0.77	1.23, *P*=0.3	NA
Omitting Jin X et al.	0.87, *P*=0.18	NA	NA	NA
Omitting Kang M et al.	0.89, *P*=0.27	1.04, *P*=0.83	NA	NA
Omitting Kim D et al.	0.88, *P*=0.26	1.03, *P*=0.88	1.25, *P*=0.32	NA
Omitting Lanthier N et al.	0.9, *P*=0.31	1.06, *P*=0.72	1.15, *P*=0.5	1.77, *P*=0.16
Omitting Laszkowska M et al.	0.9, *P*=0.32	NA	NA	NA
Omitting Leal T et al.	0.9, *P*=0.34	NA	NA	NA
Omitting Liang J et al.	0.87, *P*=0.22	1, *P*=0.99	1.14, *P*=0.54	1.46, *P*=0.42
Omitting Liu J et al.	0.86, *P*=0.14	NA	NA	NA
Omitting Livanos A et al.	0.9, *P*=0.33	NA	NA	NA
Omitting Luo S et al.	0.88, *P*=0.25	NA	NA	NA
Omitting Ma X et al.	0.87, *P*=0.19	0.98, *P*=0.9	NA	NA
Omitting Montazeri M et al.	0.87, *P*=0.19	NA	NA	NA
Omitting Moura D et al.	0.88, *P*=0.24	NA	NA	NA
Omitting Nobel Y et al.	0.89, *P*=0.27	1.03, *P*=0.87	1.19, *P*=0.37	NA
Omitting Pan L et al.	0.87, *P*=0.22	NA	NA	NA
Omitting Peng X et al.	0.85, *P*=0.13	0.97, *P*=0.84	1.1, *P*=0.64	1.42, *P*=0.44
Omitting Ramachandran P et al.	0.87, *P*=0.21	NA	NA	NA
Omitting Redd W et al.	0.89, *P*=0.28	NA	NA	NA
Omitting Renelus B et al.	0.89, *P*=0.29	NA	NA	NA
Omitting Russell B et al.	0.86, *P*=0.18	NA	NA	NA
Omitting Schettino M et al.	0.92, *P*=0.43	NA	NA	NA
Omitting Shang H et al.	0.86, *P*=0.17	0.97, *P*=0.85	NA	NA
Omitting Soares R et al.	0.9, *P*=0.32	1.08, *P*=0.66	NA	NA
Omitting Sulaiman T et al.	0.88, *P*=0.24	NA	NA	NA
Omitting Tsibouris P et al.	0.89, P=0.27	1.04, *P*=0.83	1.17, *P*=0.45	1.49, *P*=0.38
Omitting Vena A et al.	0.88, *P*=0.23	1.01, *P*=0.95	1.23, *P*=0.32	NA
Omitting Villanego F et al.	0.89, *P*=0.29	NA	NA	NA
Omitting Vrillon A et al.	0.89, *P*=0.3	NA	NA	NA
Omitting Wan Y et al.	0.86, *P*=0.15	0.96, *P*=0.78	NA	NA
Omitting Wang Z et al.	0.86, *P*=0.16	0.96, *P*=0.8	1.13, *P*=0.53	NA
Omitting Yang X et al.	0.88, *P*=0.24	NA	1.18, *P*=0.43	NA
Omitting Zhang J et al.	0.89, *P*=0.26	1.03, *P*=0.86	1.18, *P*=0.43	1.52, *P*=0.35
Omitting Zhang L et al.	0.86, *P*=0.17	0.97, *P*=0.86	1.21, *P*=0.4	1.91, *P*=0.17
Omitting Zhou F et al.	0.88, *P*=0.24	1.02, *P*=0.91	1.13, *P*=0.56	NA
Omitting Zhou Z et al.	0.88, *P*=0.23	NA	NA	NA

OR, odds ratio; GI, gastrointestinal; NA, not available

## Discussion

Several previous literatures have revealed that the GI symptoms might be associated with the prognosis with COVID-19 [7]. However, in the current meta-analysis, neither GI symptoms nor individual symptoms including diarrhea, nausea/vomiting and abdominal pain shows a significant relevance with the mortality of COVID-19 patients. Besides, the present data suggest that older age might be a significant predictor of poor prognosis in COVID-19 patients with GI symptoms. Based on the current available data, there is no convincing evidence that GI symptoms may be associated with higher risk of mortality in COVID-19 patients.

The prognosis index of COVID-19 includes several aspects such as admission to intensive care unit, low pulse oxygen saturation, development of acute respiratory distress syndrome and death of disease. A large number of previous studies investigated the relationship of GI symptoms and the severity of COVID-19, but not mortality [64–66]. This is mainly due to the limited death cases in the first few months of disease outbreak. GI symptoms have been found common in COVID-19 patients in numerous studies [67], and are considered to indicate the involvement of digestive system by virus [68]. Xiao et al. identified the infection of SARS-CoV-2 in the cytoplasm of gastric, duodenal, and rectum glandular epithelial cell by immunofluorescent staining of gastrointestinal tissues from hospitalized patients infected with SARS-CoV2 [6]. There have been views that GI symptoms might indicate a more invasive pattern of virus [7, 8, 69]. Quite a few clinical researches have observed the GI symptoms as a risk factor for disease severity of COVID-19. Jin et al. [29] found that for patients with GI symptoms (n = 74), 22.97% developed severe/critical type of disease; while for patients without GI symptoms (n = 577), only 8.14% were severe/critical type (*P* < 0.001). The meta-analysis by Mao et al. also found GI symptoms a significant risk factor for disease severity (OR = 3.97; 95% CI 1.49–10.62; *P* = 0.006) [8]. They included 4 studies to explore the influence of GI symptoms on mortality. Although they yielded an OR of 1.21, it was without statistical significance (95% CI 0.68–2.16; *P* = 0.52). The limited number of included studies and death cases (n = 29) might restrict the statistical power. However, with more abundant patients who met the endpoint in out meta-analysis, the correlation of GI symptoms in COVID-19 patients and mortality is still non-significant.

Despite the points of view highlighting the importance of GI symptoms in COVID-19, there exist arguments. In another meta-analysis by Wang et al., no significant differences were detected in the prevalence of diarrhea (OR = 1.24; 95% CI 0.90 to 1.72; *P* = 0.19) and nausea/vomiting (OR = 1.24; 95% CI 0.57 to 2.69; *P* = 0.58) between non-severe and severe COVID-19 patients [70]. They held the view that GI symptoms were not associated with the COVID-19 progression, and SARS-CoV-2-induced liver injury deserved more attention [70]. Nobel et al. proposed that gastrointestinal symptoms were associated with a more indolent form of COVID-19 based on their clinical observation [42]. Although the digestive system can be involved, most of the symptoms are mild and can be improved by supportive treatments, thus might have less impact upon disease severity. On the other hands, the respiratory tract is more commonly involved in COVID-19 and most patients died of respiratory failure. The gastrointestinal involvement might not be a prominent factor compared with other underlying diseases or respiratory failure.

There are several strengths of this meta-analysis. To the best of our knowledge, up to now this is a relatively large meta-analysis on the specific influence of GI symptoms on the mortality of COVID-19. We have included a large number of literatures, with patient population above fifty thousand and 4,955 non-survivors among them, spanning five continents. We have also excluded studies with small sample size (< 20), and most studies included in calculating the pooled OR estimates had more than 100 patients. Besides, the publication bias has been adjusted and the outcome remains the same, which make the conclusion more reliable.

Old age have been found to be independently associated with mortality in quite a few investigations [14]. As is known old age is related with increased incidence of comorbidities, cognitive impairment, dependence, and frailty. The immuno-senescence in the elderly might also lead to a different reaction against infections. The recent reports and the present meta-analysis have emphasized the differences in mortality for patients of a certain age exhibiting GI symptoms. It has been reported that adults over 60 years of age account for 96% of deaths caused by COVID-19 [71] A significant portion of COVID-19 patients have digestive symptoms, mostly at presentation. Therefore, GI symptoms should also be taken into account so as to maintain a high level of suspicion to reach an early diagnosis and set up infection control measures to improve the prognosis of elderly patients with COVID-19.

This meta-analysis has two potential limitations. As mentioned, there might exist potential patients selection bias in the age of patients with/without GI symptoms in different countries. This might lead to the discordance in the results of different study subgroups. On the other hand, currently there are no studies designed to prospectively compare the mortality of COVID-19 patients with/without GI symptoms, thus we have to include retrospective reports, which might limit the quality of evidence. Future prospective observational studies are needed to further clarify the role of GI symptoms in COVID-19.

## Conclusions

In summary, we have shown in this meta-analysis that the presence of GI symptoms is not associated with the risk of mortality in COVID-19 patients. The prognostic value of GI symptoms in COVID-19 might not be as significant as other factors such as age, concomitant underlying diseases and respiratory manifestations. Further investigations are needed to clarify the role of gastrointestinal involvement in the disease course of COVID-19, and to explore its therapeutic implications.

## Supplementary Information

**Additional 1.** Lists all the extracted data which were used to generate all the results of this study.

**Additional 2.** Contains the supplementary forest plots and the corresponding figure legends.

## Data Availability

The dataset generated and analysed during the current study is available in the Additional file 1.

## Abbreviations

COVID-19: corona virus disease 2019
GI: gastrointestinal
OR: odds ratio
NOS: Newcastle-Ottawa Scale
CI: confidence interval

## References

[1] Huang C, Wang Y, Li X, Ren L, Zhao J, Hu Y (2020). Clinical features of patients infected with 2019 novel coronavirus in Wuhan, China. Lancet.

[2] Mahase E (2020). Covid-19: WHO declares pandemic because of “alarming levels” of spread, severity, and inaction. BMJ.

[3] Remes-Troche JM, Ramos-de-la-Medina A, Manriquez-Reyes M, Martinez-Perez-Maldonado L, Lara EL, Solis-Gonzalez MA (2020). Initial Gastrointestinal Manifestations in Patients With Severe Acute Respiratory Syndrome Coronavirus 2 Infection in 112 Patients From Veracruz in Southeastern Mexico. Gastroenterology.

[4] Cholankeril G, Podboy A, Aivaliotis VI, Tarlow B, Pham EA, Spencer S (2020). High Prevalence of Concurrent Gastrointestinal Manifestations in Patients With Severe Acute Respiratory Syndrome Coronavirus 2: Early Experience From California. Gastroenterology.

[5] Qi F, Qian S, Zhang S, Zhang Z (2020). Single cell RNA sequencing of 13 human tissues identify cell types and receptors of human coronaviruses. Biochem Biophys Res Commun.

[6] Xiao F, Tang M, Zheng X, Liu Y, Li X, Shan H (2020). Evidence for Gastrointestinal Infection of SARS-CoV-2. Gastroenterology.

[7] Zheng T, Yang C, Wang HY, Chen X, Yu L, Wu ZL (2020). Clinical characteristics and outcomes of COVID-19 patients with gastrointestinal symptoms admitted to Jianghan Fangcang Shelter Hospital in Wuhan, China. J Med Virol.

[8] Mao R, Qiu Y, He JS, Tan JY, Li XH, Liang J (2020). Manifestations and prognosis of gastrointestinal and liver involvement in patients with COVID-19: a systematic review and meta-analysis. Lancet Gastroenterol Hepatol.

[9] Higgins J, Green S (2011). Cochrane handbook for systematic reviews of interventions Version 5.1. 0.

[10] Wells GA, Shea B, O’Connell D, Peterson J, Welch V, Losos M, et al. The Newcastle-Ottawa Scale (NOS) for assessing the quality of nonrandomised studies in meta-analyses. Oxford; 2000.

[11] Alizadehsani R, Sani ZA, Behjati M, Roshanzamir Z, Hussain S, Abedini N (2021). Risk factors prediction, clinical outcomes, and mortality in COVID-19 patients. J Med Virol.

[12] An P, Chen H, Ren H, Su J, Ji M, Kang J (2021). Gastrointestinal Symptoms Onset in COVID-19 Patients in Wuhan, China. Dig Dis Sci.

[13] Atalla E, Zhang R, Shehadeh F, Mylona EK, Tsikala-Vafea M, Kalagara S (2020). Clinical Presentation, Course, and Risk Factors Associated with Mortality in a Severe Outbreak of COVID-19 in Rhode Island, USA, April-June 2020. Pathogens.

[14] Caillard S, Anglicheau D, Matignon M, Durrbach A, Greze C, Frimat L (2020). An initial report from the French SOT COVID Registry suggests high mortality due to COVID-19 in recipients of kidney transplants. Kidney Int.

[15] Chadalavada P, Padbidri V, Garg R, Alomari M, Babar A, Kewan T (2020). Transaminases are Potential Biomarkers of Disease Severity in COVID-19 Patients: A Single-Center Experience. Cureus.

[16] Chen R, Yu YL, Li W, Liu Y, Lu JX, Chen F (2020). Gastrointestinal Symptoms Associated With Unfavorable Prognosis of COVID-19 Patients: A Retrospective Study. Front Med (Lausanne).

[17] Chen T, Wu D, Chen H, Yan W, Yang D, Chen G (2020). Clinical characteristics of 113 deceased patients with coronavirus disease 2019: retrospective study. BMJ.

[18] Comoglu Ş, Öztürk S, Kant A, Arslan M, Karakoc HN, Yilmaz G (2021). Evaluation of Diarrhea in Patients with COVID-19. Dig Dis.

[19] Crespo M, Mazuecos A, Rodrigo E, Gavela E, Villanego F, Sánchez-Alvarez E (2020). Respiratory and Gastrointestinal COVID-19 Phenotypes in Kidney Transplant Recipients. Transplantation.

[20] Doganci S, Ince ME, Ors N, Yildirim AK, Sir E, Karabacak K (2020). A new COVID-19 prediction scoring model for in-hospital mortality: experiences from Turkey, single center retrospective cohort analysis. Eur Rev Med Pharmacol Sci.

[21] Du H, Dong X, Zhang JJ, Cao YY, Akdis M, Huang PQ (2021). Clinical characteristics of 182 pediatric COVID-19 patients with different severities and allergic status. Allergy.

[22] Elimian KO, Ochu CL, Ebhodaghe B, Myles P, Crawford EE, Igumbor E (2020). Patient characteristics associated with COVID-19 positivity and fatality in Nigeria: retrospective cohort study. BMJ Open.

[23] Ferm S, Fisher C, Pakala T, Tong M, Shah D, Schwarzbaum D (2020). Analysis of Gastrointestinal and Hepatic Manifestations of SARS-CoV-2 Infection in 892 patients in Queens, NY. Clin Gastroenterol Hepatol.

[24] Gayam V, Chobufo MD, Merghani MA, Lamichhane S, Garlapati PR, Adler MK (2021). Clinical characteristics and predictors of mortality in African-Americans with COVID-19 from an inner-city community teaching hospital in New York. J Med Virol.

[25] Ghoshal UC, Ghoshal U, Mathur A, Singh RK, Nath A, Garg A (2020). The Spectrum of Gastrointestinal Symptoms in Patients With Coronavirus Disease-19: Predictors, Relationship With Disease Severity, and Outcome. Clin Transl Gastroenterol.

[26] Hajifathalian K, Krisko T, Mehta A, Kumar S, Schwartz R, Fortune B (2020). Gastrointestinal and Hepatic Manifestations of 2019 Novel Coronavirus Disease in a Large Cohort of Infected Patients From New York: Clinical Implications. Gastroenterology.

[27] Huang H, Zhang M, Chen C, Zhang H, Wei Y, Tian J (2020). Clinical Characteristics of COVID-19 in patients with pre-existing ILD: A retrospective study in a single center in Wuhan, China. J Med Virol.

[28] Jiang Y, Abudurexiti S, An MM, Cao D, Wei J, Gong P (2020). Risk factors associated with 28-day all-cause mortality in older severe COVID-19 patients in Wuhan, China: a retrospective observational study. Sci Rep.

[29] Jin X, Lian JS, Hu JH, Gao J, Zheng L, Zhang YM (2020). Epidemiological, clinical and virological characteristics of 74 cases of coronavirus-infected disease 2019 (COVID-19) with gastrointestinal symptoms. Gut.

[30] Kang MK, Kim KO, Kim MC, Cho JH, Kim SB, Park JG (2020). Clinical characteristics of coronavirus disease 2019 patients with diarrhea in Daegu. Korean J Intern Med.

[31] Kim D, Adeniji N, Latt N, Kumar S, Bloom PP, Aby ES (2021). Predictors of Outcomes of COVID-19 in Patients with Chronic Liver Disease: US Multi-center Study. Clin Gastroenterol Hepatol.

[32] Lanthier N, Mahiat C, Henrard S, Stärkel P, Gilard I, De Brauwer I (2021). Gastro-intestinal symptoms are associated with a lower in-hospital mortality rate in frail older patients hospitalized for COVID-19. Acta Gastroenterol Belg.

[33] Laszkowska M, Faye AS, Kim J, Truong H, Silver ER, Ingram M (2021). Disease Course and Outcomes of COVID-19 Among Hospitalized Patients With Gastrointestinal Manifestations. Clin Gastroenterol Hepatol.

[34] Leal T, Costa E, Arroja B, Goncalves R, Alves J (2021). Gastrointestinal manifestations of COVID-19: results from a European centre. Eur J Gastroenterol Hepatol.

[35] Liang J, Jin G, Liu T, Wen J, Li G, Chen L (2021). Clinical characteristics and risk factors for mortality in cancer patients with COVID-19. Front Med.

[36] Liu J, Tao L, Liu X, Yao H, Yu S, Wang Q (2021). GI symptoms and fever increase the risk of severe illness and death in patients with COVID-19. Gut.

[37] Livanos AE, Jha D, Cossarini F, Gonzalez-Reiche AS, Tokuyama M, Aydillo T (2021). Intestinal Host Response to SARS-CoV-2 Infection and COVID-19 Outcomes in Patients With Gastrointestinal Symptoms. Gastroenterology.

[38] Luo S, Deng Z, Zhang X, Pan Z, Xu H (2021). Clinical characteristics and outcomes of 2019 novel coronavirus disease patients presenting with initial gastrointestinal symptoms in Wuhan, China: A retrospective cohort study. J Gastroenterol Hepatol.

[39] Ma X, Li A, Jiao M, Shi Q, An X, Feng Y (2020). Characteristic of 523 COVID-19 in Henan Province and a Death Prediction Model. Front Public Health.

[40] Montazeri M, Maghbouli N, Jamali R, Sharifi A, Pazoki M, Salimzadeh A (2021). Clinical Characteristics of COVID-19 Patients with Gastrointestinal Symptoms. Arch Iran Med.

[41] Moura DTH (2020). Gastrointestinal Manifestations and Associated Health Outcomes of COVID-19: A Brazilian Experience From the Largest South American Public Hospital. Clin (Sao Paulo).

[42] Nobel YR, Phipps M, Zucker J, Lebwohl B, Wang TC, Sobieszczyk ME (2020). Gastrointestinal Symptoms and Coronavirus Disease 2019: A Case-Control Study From the United States. J Gastroenterol.

[43] Pan L, Mu M, Yang P, Sun Y, Wang R, Yan J (2020). Clinical characteristics of COVID-19 patients with digestive symptoms in Hubei, China: A descriptive, cross-sectional, multicenter study. Am J Gastroenterol.

[44] Peng X, Chen Y, Deng L, Liu Q, Li Q, Xiong J (2020). Clinical features of critically ill patients infected with SARS-CoV-2 outside Wuhan with and without diabetes. Int J Diabetes Dev Ctries.

[45] Ramachandran P, Onukogu I, Ghanta S, Gajendran M, Perisetti A, Goyal H (2020). Gastrointestinal Symptoms and Outcomes in Hospitalized Coronavirus Disease 2019 Patients. Dig Dis.

[46] Redd WD, Zhou JC, Hathorn KE, McCarty TR, Bazarbashi AN, Thompson CC (2020). Prevalence and Characteristics of Gastrointestinal Symptoms in Patients with SARS-CoV-2 Infection in the United States: A Multicenter Cohort Study. Gastroenterology.

[47] Renelus BD, Khoury N, Chandrasekaran K, Bekele E, Briggs WM, Jamorabo DS (2020). Hospitalized coronavirus disease-2019 (COVID-19) patients with gastrointestinal symptoms have improved survival to discharge. Dig Liver Dis.

[48] Russell B, Moss C, Papa S, Irshad S, Ross P, Spicer J (2020). Factors Affecting COVID-19 Outcomes in Cancer Patients: A First Report From Guy’s Cancer Center in London. Front Oncol.

[49] Schettino M, Pellegrini L, Picascia D, Saibeni S, Bezzio C, Bini F (2021). Clinical Characteristics of COVID-19 Patients With Gastrointestinal Symptoms in Northern Italy: A Single-Center Cohort Study. Am J Gastroenterol.

[50] Shang H, Bai T, Chen Y, Huang C, Zhang S, Yang P (2020). Outcomes and implications of diarrhea in patients with SARS-CoV-2 infection. Scand J Gastroenterol.

[51] Soares RCM, Mattos LR, Raposo LM (2020). Risk Factors for Hospitalization and Mortality due to COVID-19 in Espírito Santo State, Brazil. Am J Trop Med Hyg.

[52] Sulaiman T, Algharawi AA, Idrees M, Alzaidy RH, Faris K, Cullingford G (2020). The prevalence of gastrointestinal symptoms among patients with COVID-19 and the effect on the severity of the disease. JGH Open.

[53] Tsibouris P, Ekmektzoglou K, Agorogianni A, Kalantzis C, Theofanopoulou A, Toumbelis K (2020). Gastrointestinal involvement in COVID-19 patients: a retrospective study from a Greek COVID-19 referral hospital. Ann Gastroenterol.

[54] Vena A, Giacobbe DR, Di Biagio A, Mikulska M, Taramasso L, De Maria A (2020). Clinical characteristics, management and in-hospital mortality of patients with coronavirus disease 2019 in Genoa, Italy. Clin Microbiol Infect.

[55] Villanego F, Mazuecos A, Pérez-Flores IM, Moreso F, Andrés A, Jiménez-Martín C (2021). Predictors of severe COVID-19 in kidney transplant recipients in the different epidemic waves: Analysis of the Spanish Registry. Am J Transplant.

[56] Vrillon A, Hourregue C, Azuar J, Grosset L, Boutelier A, Tan S (2020). COVID-19 in Older Adults: A Series of 76 Patients Aged 85 Years and Older with COVID-19. J Am Geriatr Soc.

[57] Wan Y, Li J, Shen L, Zou Y, Hou L, Zhu L (2020). Enteric involvement in hospitalised patients with COVID-19 outside Wuhan. Lancet Gastroenterol Hepatol.

[58] Wang ZH, Shu C, Ran X, Xie CH, Zhang L (2020). Critically Ill Patients with Coronavirus Disease 2019 in a Designated ICU: Clinical Features and Predictors for Mortality. Risk Manag Healthc Policy.

[59] Yang X, Yu Y, Xu J, Shu H, Xia J, Liu H (2020). Clinical course and outcomes of critically ill patients with SARS-CoV-2 pneumonia in Wuhan, China: a single-centered, retrospective, observational study. Lancet Respir Med.

[60] Zhang J, Wang X, Jia X, Li J, Hu K, Chen G (2020). Risk factors for disease severity, unimprovement, and mortality in COVID-19 patients in Wuhan, China. Clin Microbiol Infect.

[61] Zhang L, Han C, Zhang S, Duan C, Shang H, Bai T (2021). Diarrhea and altered inflammatory cytokine pattern in severe coronavirus disease 2019: Impact on disease course and in-hospital mortality. J Gastroenterol Hepatol.

[62] Zhou F, Yu T, Du R, Fan G, Liu Y, Liu Z (2020). Clinical course and risk factors for mortality of adult inpatients with COVID-19 in Wuhan, China: a retrospective cohort study. Lancet.

[63] Zhou Z, Zhao N, Shu Y, Han S, Chen B, Shu X (2020). Effect of Gastrointestinal Symptoms in Patients With COVID-19. Gastroenterology.

[64] Zhu Z, Cai T, Fan L, Lou K, Hua X, Huang Z (2020). Clinical value of immune-inflammatory parameters to assess the severity of coronavirus disease 2019. Int J Infect Dis.

[65] Chen W, Lan Y, Yuan X, Deng X, Li Y, Cai X (2020). Detectable 2019-nCoV viral RNA in blood is a strong indicator for the further clinical severity. Emerg Microbes Infect.

[66] Zachariah P, Johnson CL, Halabi KC, Ahn D, Sen AI, Fischer A, et al. Epidemiology, Clinical Features, and Disease Severity in Patients With Coronavirus Disease 2019 (COVID-19) in a Children’s Hospital in New York City, New York. JAMA Pediatr. 2020;174(10):e202430. 10.1001/jamapediatrics.2020.2430 PMC7270880 32492092

[67] Cheung KS, Hung IFN, Chan PPY, Lung KC, Tso E, Liu R (2020). Gastrointestinal Manifestations of SARS-CoV-2 Infection and Virus Load in Fecal Samples From a Hong Kong Cohort: Systematic Review and Meta-analysis. Gastroenterology.

[68] Yeo C, Kaushal S, Yeo D (2020). Enteric involvement of coronaviruses: is faecal-oral transmission of SARS-CoV-2 possible?. Lancet Gastroenterol Hepatol.

[69] Musa S (2020). Hepatic and gastrointestinal involvement in coronavirus disease 2019 (COVID-19): What do we know till now?. Arab J Gastroenterol.

[70] Wang H, Qiu P, Liu J, Wang F, Zhao Q (2020). The liver injury and gastrointestinal symptoms in patients with Coronavirus Disease 19: A systematic review and meta-analysis. Clin Res Hepatol Gastroenterol.

[71] Casas-Deza D, Bernal-Monterde V, Aranda-Alonso AN, Montil-Miguel E, Julian-Gomara AB, Letona-Gimenez L (2021). Age-related mortality in 61,993 confirmed COVID-19 cases over three epidemic waves in Aragon, Spain. Implications for vaccination programmes. PLoS ONE.

