# Supplementary material for: Are gastrointestinal symptoms associated with higher risk of Mortality in COVID-19 patients? A systematic review and meta-analysis
Source: BMC Gastroenterol. 2022 Mar 7;22:106. doi: 10.1186/s12876-022-02132-0 (PMC8899790; doi:10.1186/s12876-022-02132-0)
Supplement: Supplementary file 2 — Additional 2. Contains the supplementary forest plots and the corresponding figure legends. [file 12876_2022_2132_MOESM2_ESM.docx]

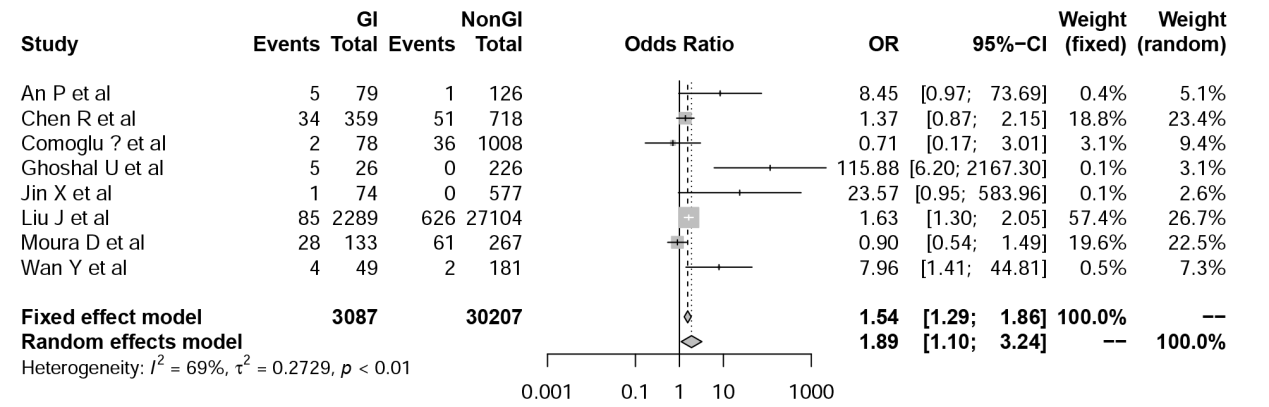


**Supplementary Figure 1**. Forest plot for odd ratio (OR) of gastrointestinal (GI) group versus nonGI group for mortality in subgroup: GI group older than non-GI group.


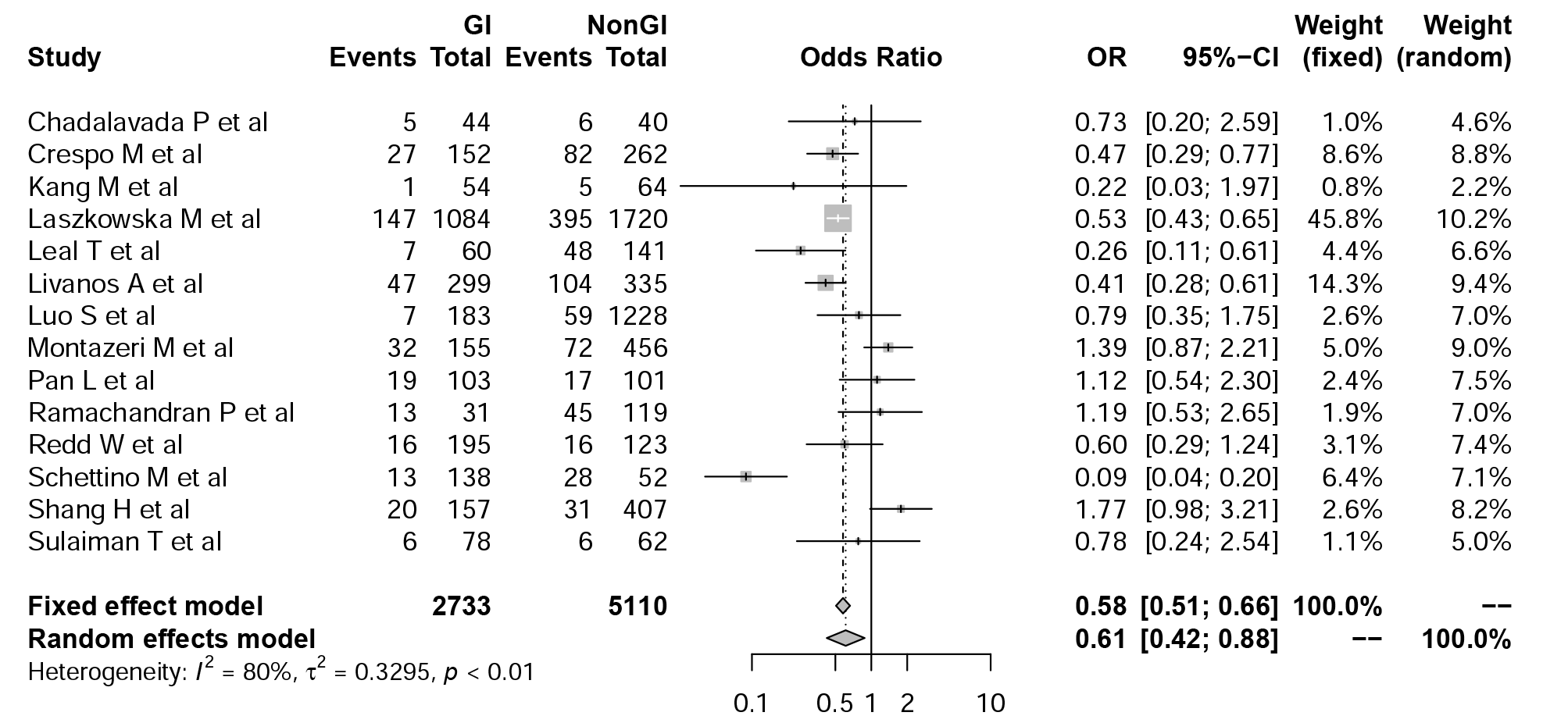


**Supplementary Figure 2**. Forest plot for odd ratio (OR) of gastrointestinal (GI) group versus nonGI group for mortality in subgroup: GI group younger than non-GI group.


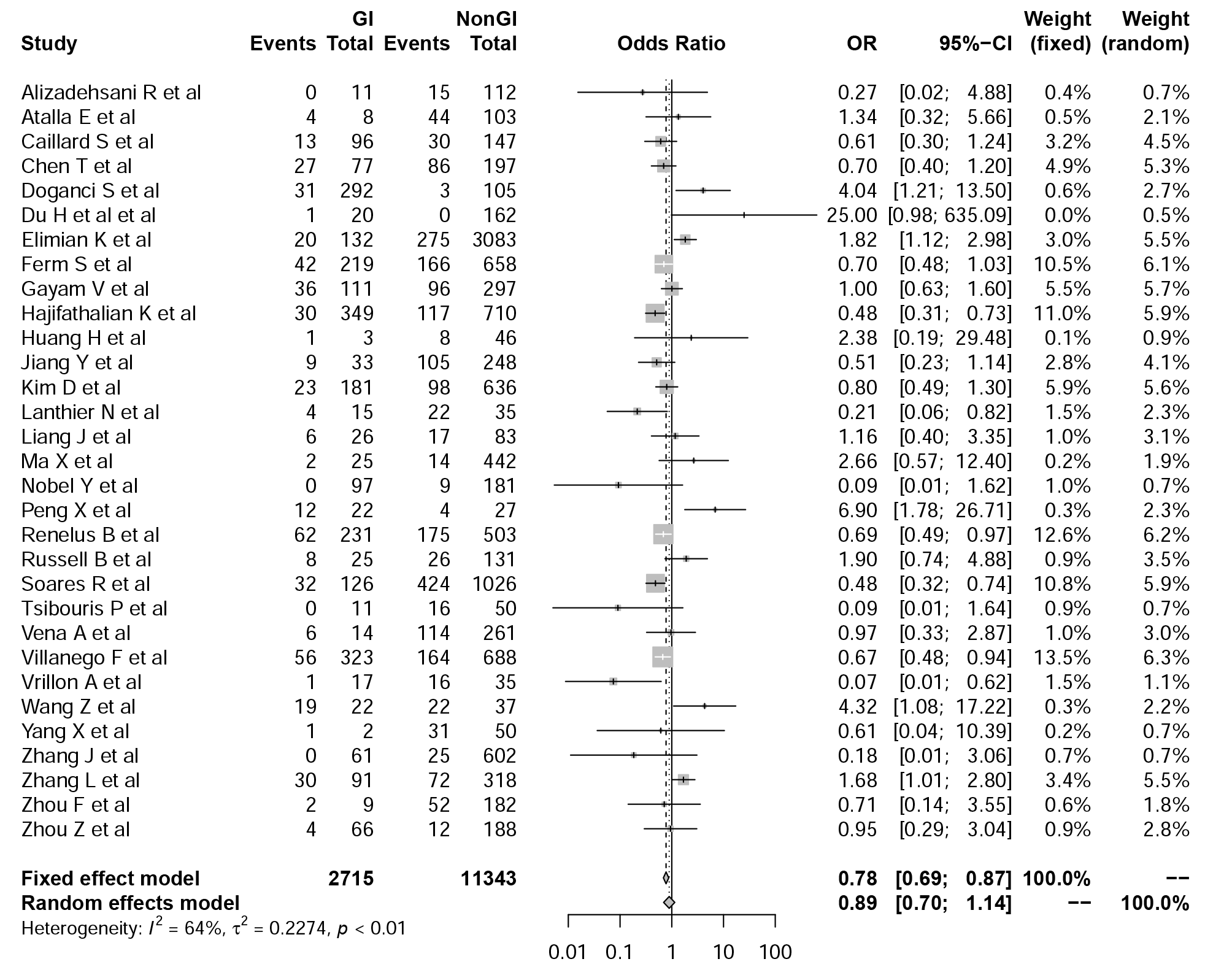


**Supplementary Figure 3**. Forest plot for odd ratio (OR) of gastrointestinal (GI) group versus nonGI group for mortality in subgroup: unknown average age of GI group and non-GI group.


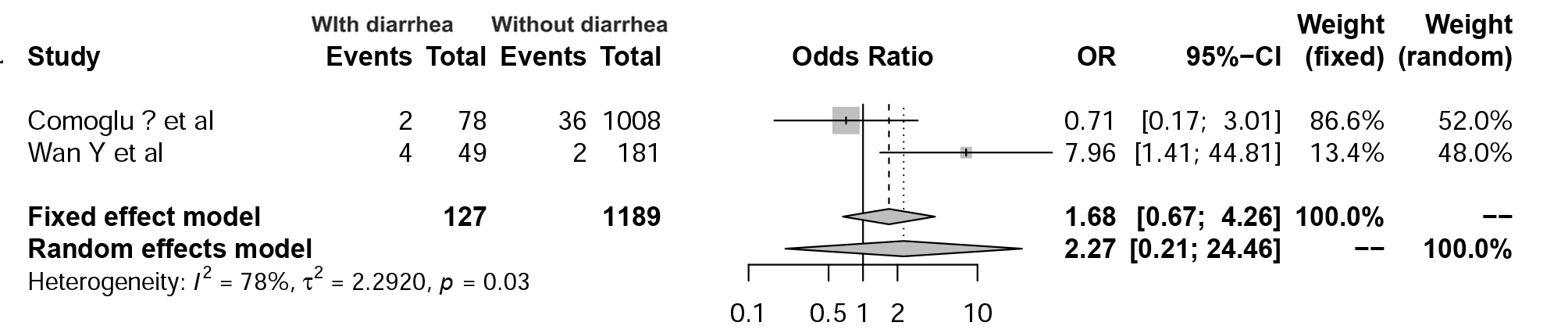


**Supplementary Figure 4**. Forest plot for odd ratio (OR) of diarrhea group versus without diarrhea group for mortality in subgroup: GI group older than non-GI group.


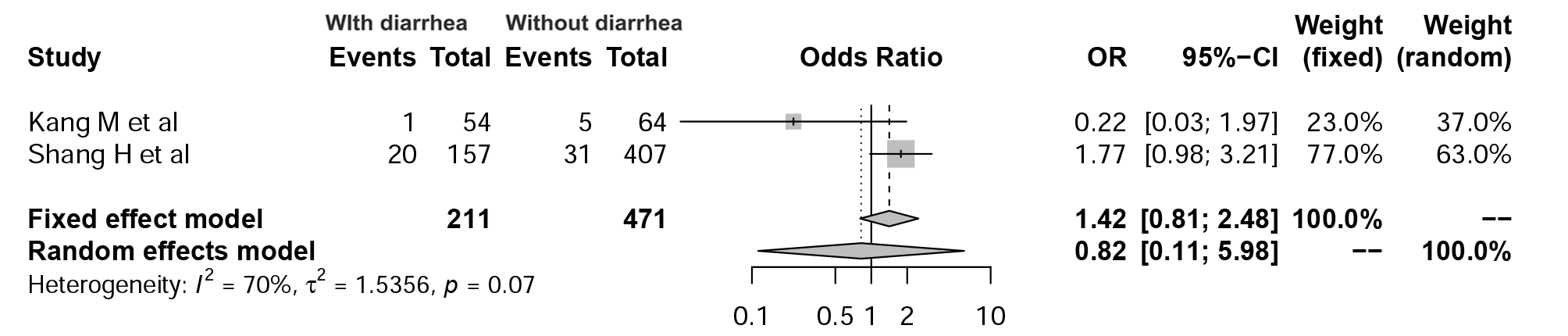


**Supplementary Figure 5**. Forest plot for odd ratio (OR) of diarrhea group versus without diarrhea group for mortality in subgroup: GI group younger than non-GI group.


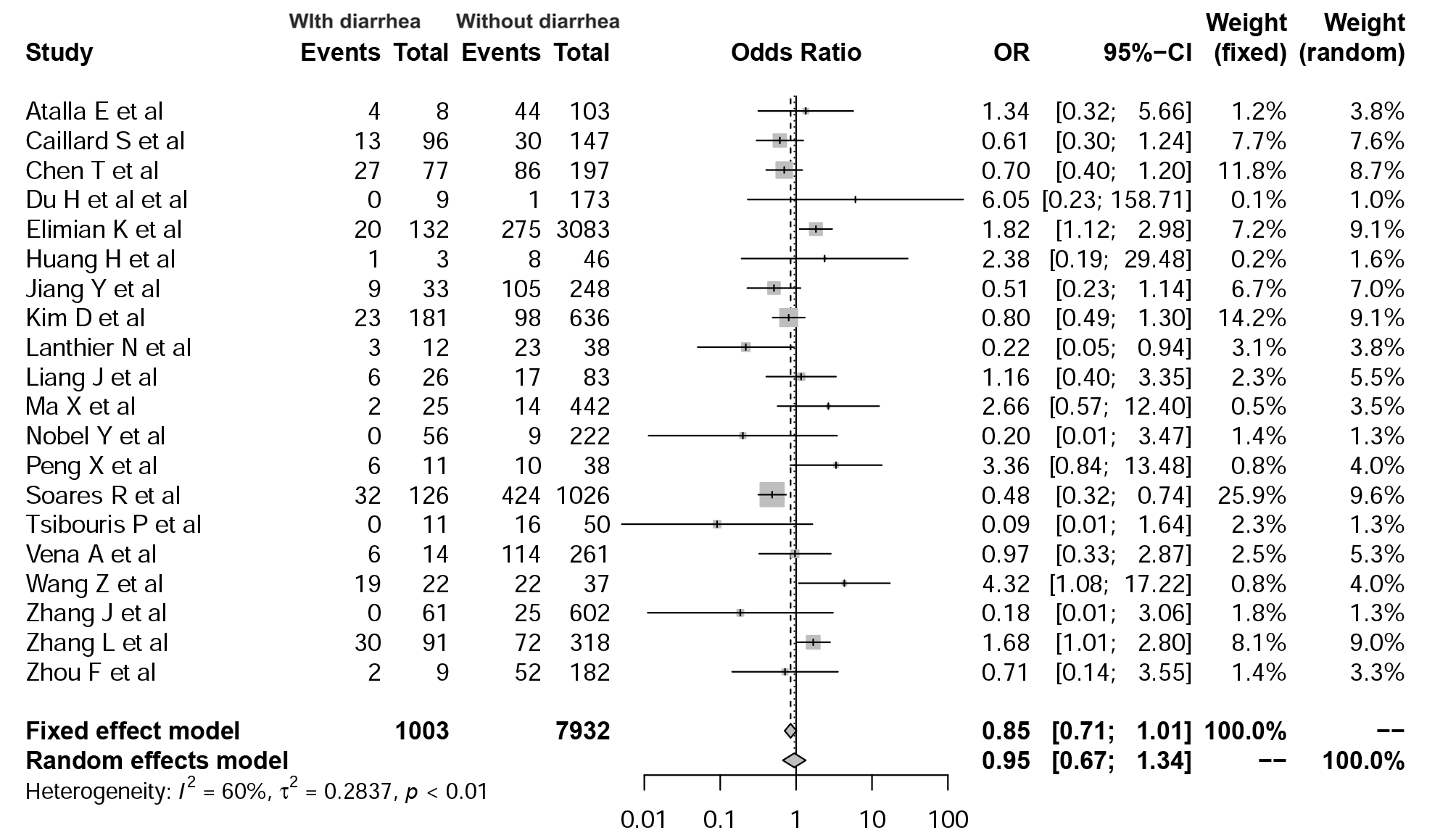


**Supplementary Figure 6**. Forest plot for odd ratio (OR) of diarrhea group versus without diarrhea group for mortality in subgroup: unknown average age of GI group and non-GI group.


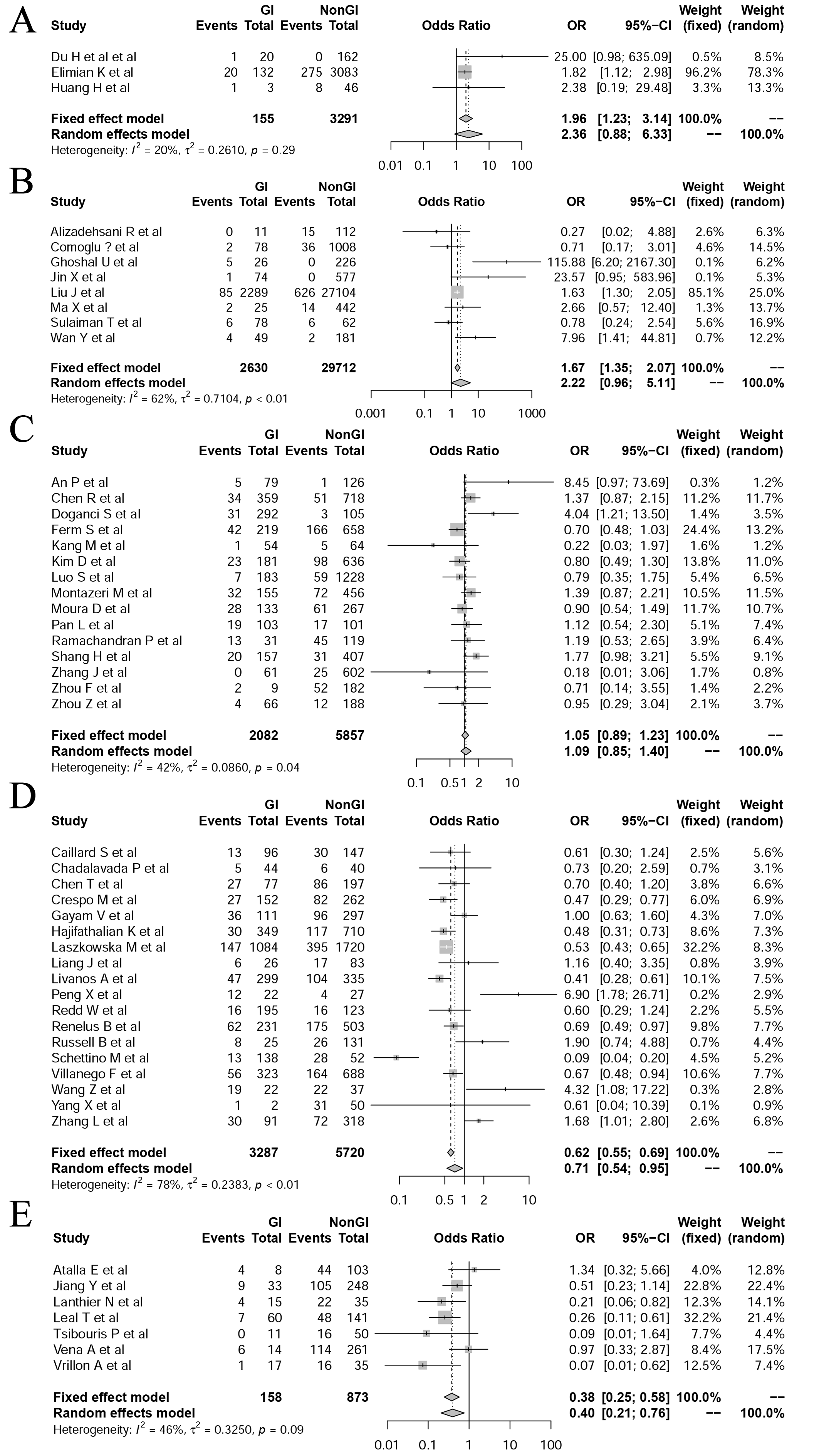


**Supplementary Figure 7**. Forest plots for odd ratio (OR) of gastrointestinal (GI) group versus nonGI group for mortality in different age stratifications: **(A)** 0~39 years old; **(B)** 40~49 years old; **(C)** 50~59 years old; **(D)** 60~69 years old; **(E)** 70 and >70 years old.
